# Preventive Effect and Molecular Mechanism of *Lactobacillus rhamnosus* JL1 on Food-Borne Obesity in Mice

**DOI:** 10.3390/nu13113989

**Published:** 2021-11-09

**Authors:** Mo Yang, Jiapeng Zheng, Xinran Zong, Xinyan Yang, Yu Zhang, Chaoxin Man, Yujun Jiang

**Affiliations:** Key Laboratory of Dairy Science, Ministry of Education, Department of Food Science, Northeast Agricultural University, Harbin 150030, China; yang1994mo@163.com (M.Y.); zjp1997447@163.com (J.Z.); zongxinran@126.com (X.Z.); 15545116996@163.com (X.Y.); jessedevil@163.com (Y.Z.); cxman@neau.edu.cn (C.M.)

**Keywords:** *Lactobacillus rhamnosus* JL1, obesity, probiotics, signaling pathway

## Abstract

Probiotics can prevent obesity and related metabolic complications. In our study, the protective effect and molecular mechanism of *Lactobacillus rhamnosus* JL1 (separated from the feces of healthy infants) on high-fat diet mice were investigated. After 10 weeks of dietary intervention with *L. rhamnosus* JL1 intervention, the body weight of the JL1 group (23.78 g) was significantly lower than that of the HFD group (26.59 g, *p* < 0.05) and the liver index was reduced. Serum biochemical analysis showed that the TC, TG and LDL-C contents of JL1 group mice were significantly decreased (*p* < 0.05). Histological images of the mice livers showed that the degree of lipid action and damage of hepatic cells were improved. *L. rhamnosus* JL1 activated the AMPK pathway, and reduced the gene expression of PPAR-γ, LXR-α and SREBP-1C. In addition, the protein expression of PPAR-γ and LXR-α were reduced. After dietary intervention with *L. rhamnosus* JL1, the concentration of acetic acid, propionic acid, and butyric acid were increased significantly, especially the concentration of butyric acid, which was 63.16% higher than that of the HFD group (*p* < 0.05). In conclusion, this study provided a theoretical reference for the development and application of probiotics derived from healthy infant feces in health products and functional foods.

## 1. Introduction

Obesity seriously threatens human health. Chronic diseases such as tumours and diabetes are all related to obesity. Therefore, the prevention and treatment of metabolic diseases must be fully considered in their association with obesity [1]. Epidemiological evidence has confirmed that high-fat diets are closely associated with increased susceptibility to diseases such as hypertension, hyperlipidemia, and atherosclerosis [2]. Excessive fat intake increases the risk of hyperlipidemia [3]. Currently, more and more evidence shows that excessive dietary fat may enhance intestinal permeability to varying degrees, increasing inflammation [4]. Therefore, effective prevention of obesity is vital to reduce the occurrence of cardiovascular and cerebrovascular diseases. Body weight is related to eating habits, healthy diet, physical activity, and genes. Obesity is becoming more and more serious, and bariatric surgery is applied in some cases. Medicines and surgery may bring adverse effects, so it is vital to develop healthy long-term eating habits [5]. Probiotics may be able to alleviate obesity caused by high-fat and high-sugar diets; they are a potential solution for the treatment of lipid metabolism disorders caused by obesity in recent years.

Probiotic intervention is an important tool for improving the health of hosts. Lactic acid bacteria are widely found in food, the environment and the intestinal tract [6]. Among them, *Lactobacillus* has been widely used in functional foods for a long time because of its safe and effective properties [7]. Evidence suggests that supplementing with probiotics can significantly reduce obesity-related indicators. Both single and multi-bacterial species can significantly reduce body weight, TNF-α, insulin, total cholesterol, and low density lipid-cholesterol levels [8]. Probiotics can significantly reduce the fasting blood sugar of patients, and their effects are better than those obtained in patients without insulin therapy [9]. Probiotics can improve insulin sensitivity, reduce lipid levels and insulin resistance, and further alleviate cardiac dysfunction in mice on a high-fat diet [10]. Genomics and metagenomics technologies reveal that some lactic acid bacteria can colonize human intestines due to their probiotic properties, helping to reduce the risk of metabolic syndromes such as diabetes, obesity and other diseases [11].

*Lactobacillus rhamnosus* is a kind of *lactic acid bacteria,* and is one of the most widely studied probiotics. *L. rhamnosus* has anti-allergy [12], anti-anxiety and anti-depressant effects [13]. *L. rhamnosus* GG can inhibit fatty acid metabolism and absorption in the mice intestine by competing with the host intestine for fatty acids, inhibiting liver fat accumulation, and thereby preventing the occurrence of fatty liver [14]. Through the top-down strategy, probiotics can be screened in the intestines of healthy individuals to develop precision probiotics [15]. In our previous study, *L. rhamnosus* JL1 was separated from the feces of healthy infants. We have completed whole genome sequencing of the strain, and its COG functional clustering gene distribution ratio showed that the genes controlling lipid metabolism and carbohydrate transport accounted for 13.05% of the total number of genes. Animal experiments showed that it had a good anti-inflammatory effect [16].

However, due to the large differences between different strains, whether *L. rhamnosus* JL1 can effectively prevent obesity or reduce blood lipids remains to be studied, and its molecular mechanisms need to be explored. This study will establish a mouse model induced by a high-fat diet supplemented with *L. rhamnosus* JL1 from the feces of healthy infants. The preventive effects and molecular mechanisms of *L. rhamnosus* JL1 on lipid metabolism will be determined, with a view to developing precise probiotics. The study provides a theoretical basis for *L. rhamnosus* JL1 as a high value probiotic product and functional food.

## 2. Materials and Methods

### 2.1. Materials

*L. rhamnosus* JL1 was previously isolated from infant feces and stored at −80 °C until use. *L. rhamnosus* JL1 was cultured at 37 °C for 18 h in Man Rogosa Sharp medium (Hope Technology Co., Ltd., Qingdao, China). Kits for TC (total cholesterol), TG (triglyceride), LDL-C (low density lipid-cholesterol) and HDL-C (high density lipid-cholesterol) were purchased from Jiancheng Institute of Bioengineering (Nanjing, China). IL-1β, IL-6, TNF-α and IFN-γ enzyme-linked immunosorbent assay kits were purchased from Huijia Biological Technology, (Xiamen, China). BCA Protein Assay Kits were purchased from Solarbio Science and Technology Co., Ltd. (Beijing, China). Other reagents were obtained from Sino pharm Co., Ltd. (Beijing, China).

### 2.2. Cholesterol-Lowering Ability of L. rhamnosus JL1 In Vitro

The activated strains were respectively inoculated into Man Rogosa Sharp liquid medium containing 100 μg/mL of water-soluble cholesterol (filter-sterilized) at 5% inoculum, and cultivated at 37 °C for 24 h; the degradation rate of cholesterol was measured and calculated every 3 h [17].

### 2.3. Animal Groups and Feeding

Twenty four C57BL/6 male mice (20 ± 2 g, 6-weeks-old) were purchased from Weitong Lihua Laboratory Animal Technology Co., Ltd. (Beijing, China). The animal experiments in this study were allowed by the Laboratory Animal Welfare and Ethics Committee of Northeast Agricultural University (NEAU-2020-08-3001-30). The growth conditions for mice were temperatures of 22 ± 2 °C, a relative humidity of 55 ± 10%, and a 12 h light/dark cycle. After 7-days of administration of a normal diet, the mice were randomly divided into 3 groups (8 mice per group): NC group (Normal diet), HFD group (High-fat diet), and JL1 group (High-fat diet with 10^9^ CFU/mL *L. rhamnosus* JL1). The composition of the experimental diets was shown in Table 1.

The experimental mice were fed for 10 weeks. On the last day of the experiment, eyeball blood was taken from the mice after fasting for 8 h; then, the serum was separated then stored at −20 °C for later use. The body weight of experimental mice was recorded every week. The livers were quickly removed from the mice, washed with chilled normal saline, dried with filter paper, and weighed, then stored at −80 °C until use. The liver index was calculated according to the following formula:

$$\mathrm{Liver}\;\mathrm{index}(\%)=\frac{\mathrm{Liver}\;\mathrm{weight}\;(g)}{\mathrm{Body}\;\mathrm{weight}\;(g)}\times 100\%$$

### 2.4. Determination of Blood Lipid Levels of Mice Serum

The collected mouse blood was placed at room temperature for 2 h, then centrifuged at 4 °C, 3000 r/min for 10 min, and the serum was collected. The blood lipid levels were detected according to the kits.

### 2.5. Determination of Inflammatory Cytokines of Mice Serum

The inflammatory cytokine levels in serum were determined by IL-1β, IL-6, TNF-α and IFN-γ enzyme-linked immunosorbent assay kits.

### 2.6. Liver Histological Analysis

The liver tissue pieces were fixed in a solution of 10% neutral formaldehyde, trimmed to a size of 0.5 × 0.5 × 0.5 cm^3^, dehydrated, cleared and embedded in paraffin [18]. Lipid deposition and inflammation of hepatocytes were observed by an optical microscope (Olympus, Tokyo, Japan).

### 2.7. Determination of the mRNA Expression in Mouse Liver by RT-PCR

The cycling conditions and calculation methods of RT-PCR were carried out according to Sun’s method [19]. Total RNA from mouse livers was extracted. Reverse transcription of RNA into cDNA was carried out according to the reverse transcription kit operating instructions. The primers used in the experiment are listed in Table 2.

### 2.8. Determination of Protein Expression in Mouse Liver by Western Blotting

Lipid metabolism-related protein expression levels in liver tissues were measured by western blotting according to Li’s method, with slight modifications [16]. Total protein was extracted by RIPA total protein lysate. After a 30 min ice bath, the homogenate was completely lysed, and centrifuged at 13,000× *g* at 4 °C for 5 min. Total protein was measured by a BCA Protein Assay Kit. The same concentration of protein was obtained by SDS-PAGE and transferred to a PVDF membrane. The transferred membrane was added to the blocking solution and blocked at room temperature for 1 h. The blocking solution was removed and the diluted primary antibody added at 4 °C overnight. The diluted primary antibody was recovered and washed three times with TBST for 5 min each time. The diluted secondary antibody was added, incubated at room temperature for 30 min, and washed four times with TBST on a shaker at room temperature, for 5 min each time.

### 2.9. Analysis of SCFAs in the Faeces

The faeces of the mice were collected and stored at −80 °C. Then, the mice faeces were freeze-dried and weighed. The content of SCFAs in faeces was determined according to Wang’s method [20].

### 2.10. Statistical Methods

SPSS statistical software (23.0 version) was used for analysis. The data were expressed by the mean ± standard deviation (mean ± SD), and the differences between different groups were determined by one-way ANOVA. Differences were considered statistically significant with *p* < 0.05.

## 3. Results

### 3.1. Cholesterol-Lowing Ability of L. Rhamnosus JL1 In Vitro

**Figure 1:**
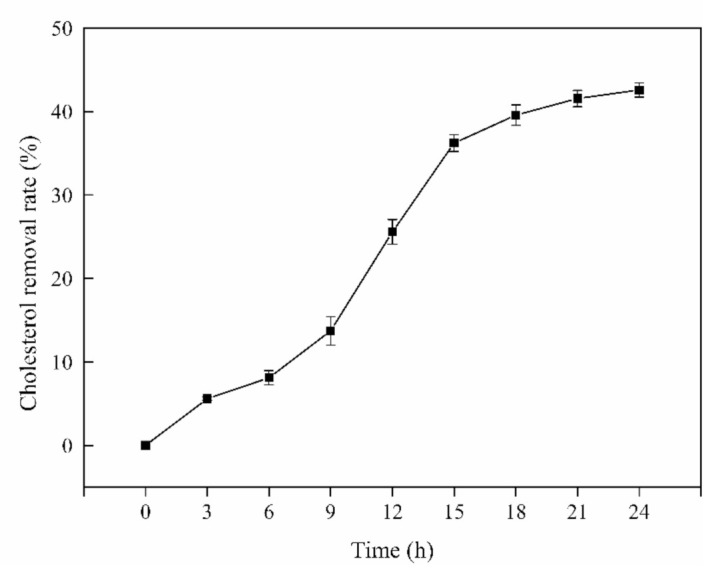
Cholesterol-lowing ability of *L. rhamnosus* JL1 in vitro. Error bars indicate standard deviation (±SD).

*L. rhamnosus* JL1 had a certain ability to remove cholesterol; the cholesterol-lowering ability of *L. rhamnosus* JL1 in vitro was shown in Figure 1. During the cultivation of *L. rhamnosus* JL1, the cholesterol removal rate changed until it became stable after 18 h. As the time of in vitro culture increased, the cholesterol removal rate of *L. rhamnosus* JL1 gradually increased. The final cholesterol removal rate of *L. rhamnosus* JL1 was 42.58%. This showed that *L. rhamnosus* JL1 had the effect of lowering cholesterol in vitro. Whether *L. rhamnosus* JL1 can exert health benefits on the host, we further determined through the animal models.

### 3.2. Effect of L. rhamnosus JL1 on Body Weight and the Liver Index of Mice

The initial body weight of the mice was between 18–22 g, and there was no significant difference among the three groups. The specific results of body weight are shown in Table 3. During the rearing period, the body weight of the three groups increased. Body weight is the most intuitive indicator reflecting food-borne obesity in mice. The results showed that the HFD group had the heaviest body weight, while the NC group was the lightest. The body weight of mice in the HFD group exceeded that of the NC group by 12.59% after a 5-week high-fat diet. After 10 weeks, the body weight of mice in the JL1 group (23.78 ± 0.84 g) was significantly lower than the body weight of the HFD group (26.59 ± 1.17 g) (*p* < 0.05).

**Figure 2:**
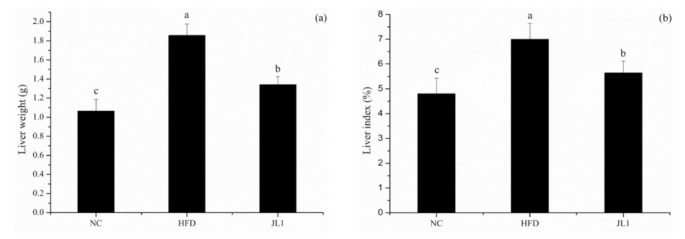
Effects of *L. rhamnosus* JL1 on the liver weight (**a**) and liver indexes (**b**) of mice. Each value represents the mean of 8 mice, and error bars indicate standard deviation (±SD). Different letters denote significant differences between different groups of mice (*p* < 0.05).

After 10 weeks of continuous intervention in each group of mice, the liver indexes of each group of mice were calculated (Figure 2). Compared with the NC group (4.80 ± 0.63%), the liver indexes of the mice in the HFD group (7.00 ± 0.64%) were significantly increased (*p* < 0.05). Moreover, compared with the HFD group, the liver indexes of the mice in the JL1 group (5.65 ± 0.47%) were significantly lower (*p* < 0.05).

### 3.3. Effect of L. rhamnosus JL1 on the Levels of Serum Lipids in Mice

After diet intervention, the serum TC contents of mice in the JL1 group (4.98 ± 0.68 mmol/L) were significantly lower than those of the HFD group (6.09 ± 0.73 mmol/L; *p* < 0.05). Compared with the HFD group, the serum TG and LDL-C content of the JL1 group decreased, respectively (Table 4). Compared with the HFD group, the serum LDL-C content of the JL1 group was decreased, and the HDL-C content was increased (*p* < 0.05), but *L. rhamnosus* JL1 cannot restore blood lipids to normal levels.

### 3.4. Cholesterol-Lowering Ability of L. rhamnosus JL1 In Vitro

In order to explore the effect of *L. rhamnosus* JL1 on mice, the contents of inflammatory cytokines (Table 5) in the serum of mice were analyzed.

The high-fat diet increased the serum levels of IL-1β (16.98 ± 2.00 ng/L), IL-6 (45.76 ± 9.21 ng/L), INF-γ (185.86 ± 19.16 ng/L) and TNF-α (187.20 ± 19.63 ng/L) of HFD mice. However, the levels of IL-1β (24.17 ± 5.08 ng/L), IL-6 (49.48 ± 11.65 ng/L), IFN-γ (227.80 ± 11.28 ng/L) and TNF-α (257.60 ± 29.79 ng/L) in the JL1 group were significantly reduced, but still higher than those in the NC group. This showed that *L. rhamnosus* JL1 can not only improve the lipid metabolism disorder of high-fat diet mice, but also reduce inflammation caused by excessive fat accumulation.

### 3.5. Liver Histological Image of Mice

**Figure 3:**
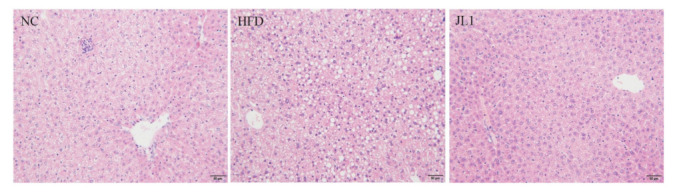
Image of liver section (scale bar = 50 μm).

It can be seen from Figure 3 that the liver lobules of the NC group had a clear complete structure, slightly hexagonal, and the liver cells were arranged radially around the central vein. There was no obvious degeneration such as necrosis or fat accumulation. There was no dilation or congestion in hepatic sinusoids, no inflammation or fibrous tissue hyperplasia in the portal area. The hepatocyte arrangement in the HFD group was disordered and the steatosis was obvious. There were lipid droplet vacuoles of different sizes in hepatocytes, some of which were larger than the liver nucleus. Inflammatory cell foci were found in mice liver tissues. The degree of hepatocellular lesions in the JL1 group was higher than that in NC group; there was no obvious necrosis and slight steatosis. No inflammatory cell infiltration was found, and no fibrous tissue proliferation was found.

### 3.6. Effect of L. rhamnosus JL1 on the Expression of Genes and Proteins in Mice

**Figure 4:**
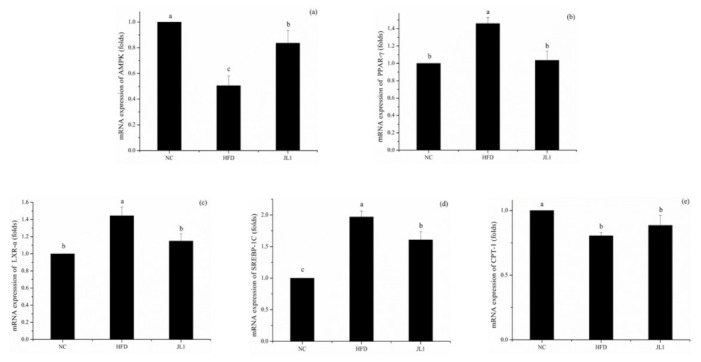
Effects of *L. rhamnosus* JL1on mRNA expression of mice liver. (**a**) mRNA expression of AMPK, (**b**) mRNA expression of PPAR-γ, (**c**) mRNA expression of LXR-α, (**d**) mRNA expression of SREBP-1C, (**e**) mRNA expression of CPT-1. Each value represents the mean of 8 mice, and error bars indicate standard deviation (±SD). Different letters denote significant differences between different groups of mice (*p* < 0.05).

**Figure 5:**
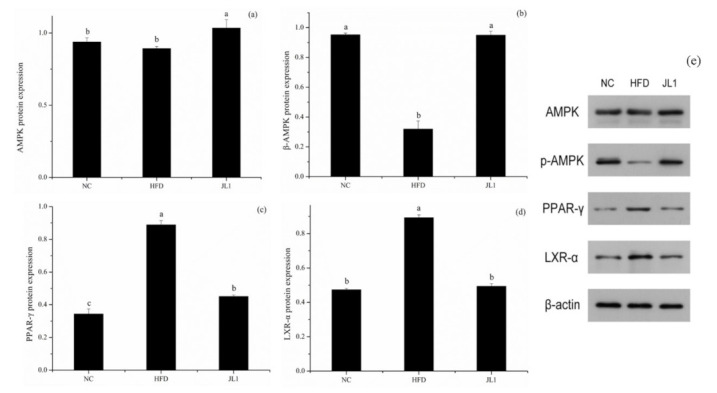
Effects of *L. rhamnosus* JL1on protein expression of mice liver. (**a**) AMPK protein expression, (**b**) p-AMPK protein expression, (**c**) PPAR-γ protein expression, (**d**) LXR-α protein expression, (**e**) Representative western blot for AMPK, p-AMPK, PPAR-γ and LXR-α. Each value represents the mean of 8 mice, and error bars indicate standard deviation (±SD). Different letters denote significant differences between different groups of mice (*p* < 0.05).

The lipid metabolism-associated gene and protein expression of experimental mice after diet intervention are shown in Figure 4 and Figure 5. In the case of long-term intake of a high-fat diet, *L. rhamnosus* JL1 can prevent food-borne obesity and reduce lipid accumulation in mice.

As shown in Figure 4, compared with the NC group, the gene expression of AMPK in the HFD group was significantly decreased (*p* < 0.05). The gene expression of AMPK in the JL1 group was significantly higher than that in the HFD group (*p* < 0.05), indicating that *L. rhamnosus* JL1 reactivated the expression of AMPK, thereby restoring the balance of lipid metabolism. Compared with the NC group, the mRNA expression of PPAR-γ, LXR-α and SREBP-1C in the HFD group increased significantly (*p* < 0.05), but the mRNA expression of PPAR-γ, LXR-α and SREBP-1C in the JL1 group decreased compared with the HFD group.

Compared with the NC group, the protein expression levels of AMPK and *p*-AMPK in the liver tissues of the HFD group decreased (*p* < 0.05), and the protein expression levels of PPAR-γ and LXR-α increased (*p* < 0.05). Compared with the HFD group, the expression levels of AMPK and *p*-AMPK protein in the liver tissues of mice in the JL1 group increased (*p* < 0.05), and the protein expression levels of PPAR-γ and LXR-α decreased (*p* < 0.05).

### 3.7. Effect of L. rhamnosus JL1 on the Concentration of SCFAs

**Figure 6:**
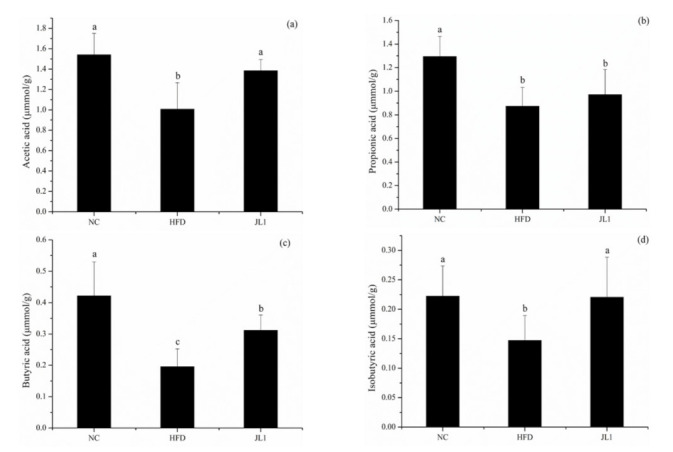
Effects of *L. rhamnosus* JL1on intestinal SCFA concentrations. (**a**) acetic acid; (**b**) propionic acid; (**c**) butyric acid; (**d**) isobutyric acid concentrations in the mice faeces. Each value represents the mean of 8 mice, and error bars indicate standard deviation (±SD). Different letters denote significant differences between different groups of mice (*p* < 0.05).

In order to further explore the mechanism of *L. rhamnosus* JL 1 in resisting high-fat diet-induced obesity, the levels of SCFAs in the faeces of each group of mice were analysed, and the results were shown in Figure 6. The high-fat diet resulted in a significant decrease in the concentration of the main SCFAs in the faeces of mice. After dietary intervention with *L. rhamnosus* JL1, the concentrations of acetic acid, propionic acid, and butyric acid were significantly increased—especially the concentration of butyric acid, which was 63.16% higher than that of the HFD group (*p* < 0.05).

## 4. Discussion

Obesity is closely related to the occurrence of chronic metabolic diseases. People’s increased energy intakes, decreased movement, and unhealthy lifestyles have led to a continuously increased incidence of obesity. The abnormal glucose and lipid metabolism caused by foodborne obesity has increased the risk of chronic diseases such as cardiovascular and cerebrovascular diseases, diabetes, and hypertension [21]. These chronic metabolic diseases have further contributed to the aggravation of obesity. The World Health Organization defines probiotics as a kind of bacterium that can adhere to and colonize the surface of the intestine, and when they reach an effective order of magnitude, they have a beneficial effect on the host [22]. As the role of gut microbiota in human health and diseases has attracted more and more attention from scientific researchers, some probiotics have been discovered to potentially reduce serum cholesterol and triglyceride levels. *L. rhamnosus* has a strong tolerance to digestive fluids such as gastric acid and bile, and can protect the intestinal barrier by adhering to intestinal epithelial cells [23]. The cholesterol removal rate of *L. rhamnosus* JL1 was 42.58% here, indicating that it may have the effect of lowering blood lipids in the host.

The liver index is an important indicator to measure the growth status of mice. Long-term consumption of a high-fat diet can induce liver injury and increase hepatic lipid accumulation [24]. When lipid metabolism is disordered, the liver is prone to steatosis, resulting in an increase in liver mass. Therefore, changes in liver coefficient can reflect the steatosis of the liver in mice. In this study, after dietary intervention by *L. rhamnosus* JL1, the body weight, liver weight and liver indexes of mice in the JL1 group were significantly lower than those in the HFD group.

Long-term high-fat diets can cause lipid metabolism disorders in mice. Blood lipids are the primary indicator for judging the blood lipid levels of mammals and humans. Common indicators for judging blood lipids include TC, TG, LDL-C and HDL-C. TC, TG and LDL-C are risk factors for coronary heart disease and atherosclerosis [25], while HDL-C can transport cholesterol and TG from surrounding tissues to the liver, and finally eliminate them through liver receptors [26]. At the same time, the HDL receptor activity is enhanced, which can transport more TC in the blood to the liver for catabolism, reducing the content of serum LDL-C and increasing the content of HDL-C to achieve the effect of regulating lipid metabolism [27]. Low-density lipoprotein cholesterol is the main component of atherosclerotic plaques and one of the causes of atherosclerotic vascular disease [3]. Dyslipidemia increases the global disease burden, and elevated plasma LDL-C levels are the main risk factors for cardiovascular and cerebrovascular diseases [28]. Supplementing probiotics can improve the lipid metabolism of obese or overweight people, and can significantly reduce TC and LDL levels especially [29]. The mechanisms of probiotics regulating lipid metabolism mainly include reducing the absorption of lipids in organisms, reducing fat intake, and inhibiting the activity of key enzymes in fatty acid synthesis and metabolism. *L. rhamnosus* JL1 can reduce the risk of atherosclerosis by reducing the content of TC, TG and LDL-C. Our results were similar to those of Yooa et al., in which *L. plantarum* KY1 and *L. curvatus* HY7601 administered to mice on a high-fat diet for 9 weeks could effectively reduce TG content in the liver and alleviate the development of non-alcoholic fatty liver disease [30].

Researchers have proposed an immunometabolic theory in recent years in which excessive fat cells trigger a chronic inflammatory response, leading to the deposition of fat in multiple organs and tissues, resulting in simple obesity. Obese patients are often accompanied by low-grade chronic inflammation, mainly manifested by increased systemic inflammation markers. IL-6 and TNF-α are classic obesity-related inflammatory factors [31]. A large number of oxygen free radicals are produced under the action of steatosis and lipid peroxidation, which will cause oxidative stress and endoplasmic reticulum stress, and further stimulate the liver Kupffer cells to produce a large amount of IL-6, IL-lβ, and TNF-α inflammatory factors, aggravating liver cell damage. *L. rhamnosus* JL1 can effectively inhibit the production of inflammatory factors such as IL-6, IL-lβ, TNF-α and IFN-γ.

AMPK is an important upstream gene that regulates the balance of lipid metabolism [32]. AMPK can inhibit fatty acid and cholesterol synthesis and promote fatty acid oxidation [33]. *L. rhamnosus* JL1 can reactivate the expression of AMPK and restore the balance of lipid metabolism. Activation of AMPK can inhibit the production of TNF-α and IL-6 [34]. This view supports the theory that *L. rhamnosus* JL1 can reduce TNF-α and IL-6 increased by excessive fat intake by activating the AMPK pathway. Scholars have found that *Lactobacillus plantarum* can improve liver lipid metabolism disorders in mice and inhibit oxidative stress through the AMPK/Nrf2 pathway to improve liver inflammation [35].

Adipogenesis is a complex transcriptional cascade involving the sequential activation of PPAR-γ, LXR-α, and SREBP-1C, which helps to activate the transcription of genes related to the adipocyte phenotype. PPAR-γ is a gene that controls fat synthesis, and when reduced it suppresses hepatic lipogenesis [36]. PPAR-γ is a member of the peroxisome proliferator-activated receptor family, mainly distributed in the liver and adipose tissue, and plays a vital role in regulating glucose and lipid metabolism and anti-inflammatory development. PPAR-γ upregulates the expression of ABCA1 and SR-B1 by activating downstream effector molecules. PPAR-γ also promotes the outflow of cholesterol from macrophages [37]. Liver X receptor (LXR), as a non-steroid hormone receptor, is a metabolic nuclear receptor that maintains the balance of cholesterol and lipid metabolism by regulating the expression of target genes [38]. LXR-α is distributed in large quantities in the liver and has the effect of regulating cholesterol metabolism. It promotes reverse cholesterol transport by regulating the downstream gene ABCA1 [39]. This study revealed that the lipid-lowering mechanism of *L. rhamnosus* JL1 was achieved by down-regulating the protein expression levels of PPAR-γ, LXR-α and SREBP-1C, causing cholesterol to be metabolized and excreted from the mice.

Tissue inflammation increases after mice become obese; supplementation of SCFAs can alleviate obesity and inflammation in mice [40]. The increase of butyric acid content may inhibit inflammation in the body, thereby reducing the content of inflammatory factors such as IL-6. SCFAs play a beneficial role in the metabolism and function of adipose tissue, skeletal muscle, and liver matrix, helping to improve insulin sensitivity [41]. SCFAs have important regulatory functions in intestinal homeostasis, and adipose tissue and liver metabolism, thereby preventing the development of obesity, type 2 diabetes and non-alcoholic fatty liver disease [42]. Studies have also shown that the intervention of probiotics can affect SCFA content. For example, *L. plantarum* HAC01 can increase the abundance of *Allobaculum* in the intestines of mice. *Allobaculum* is one of the genera producing SCFAs and is negatively correlated with weight gain [43]. SCFAs can enter the systemic circulation, and therefore affect the body’s metabolism and the function of peripheral tissues. SCFAs improve obesity by inhibiting the expression of PPAR-γ in adipose tissue [44]. We speculate that *L. rhamnosus* JL1 can inhibit the expression of key lipid metabolism genes such as PPAR-γ in liver tissues by increasing the level of SCFAs to improve obesity, while the mechanism of how *L. rhamnosus* JL1 affects the level of SCFAs still needs to be further explored. This study showed that there was a certain relationship between *L. rhamnosus* JL1 and body weight and blood lipid levels. On the one hand, *L. rhamnosus* JL1 may activate AMPK protein expression in the mouse liver, thereby regulating the PPAR-γ signaling pathway to reduce liver lipid accumulation. On the other hand, *L. rhamnosus* JL1 may regulate the content of SCFAs to relieve inflammation and improve lipid metabolism disorders. The effect of *L. rhamnosus* JL1 in reducing body weight and blood lipids may be related to inhibiting peroxidation damage and reducing cholesterol synthesis, but the specific mechanism needed further research.

## 5. Conclusions

A high-fat diet increased the body weight and blood lipids of mice. The cholesterol removal rate of * L. rhamnosus* JL1 was 42.58% in vitro. *L. rhamnosus* JL1 had a good effect on reducing blood lipids and lowering weight. After 10 weeks of diet intervention, the body weights of the JL1 group were lower than those of HFD group, indicating that *L. rhamnosus* JL1 could control the food-borne obesity of mice. The intervention of *L. rhamnosus* JL1 significantly reduced the content of TC, TG and LDL-C in the blood lipids of mice. In addition, the intervention of *L. rhamnosus* JL1 reduced the liver indexes of mice, which could effectively regulate liver lipid metabolism disorders. *L. rhamnosus* JL1 reduced the content of IL-1β, IL-6, TNF-α, IFN-γ significantly. The liver pathological section showed that the degree of hepatocyte lipidosis of the JL1 group was reduced and the damaged cells were reduced. *L. rhamnosus* JL1 increased AMPK mRNA expression and protein expression significantly. *L. rhamnosus* JL1 also increased the content of SCFAs in the intestines of mice, especially butyric acid. *L. rhamnosus* JL1 can prevent obesity by inhibiting fat synthesis and reducing inflammation. This research has great significance for the development of *L. rhamnosus* JL1 and probiotics from healthy infant feces.

## Figures and Tables

**Table 1:** Treatment for Experimental mice.

Group	Diet	Treatment
NC (Normal control)	Normal diet	0.2 mL PBS per day
HFD (High-fat diet)	High-fat diet	0.2 mL PBS per day
JL1 (*L. rhamnosus* JL1)	High-fat diet	0.2 mL PBS + 10^9^ CFU/mL *L. rhamnosus* JL1 per day

Normal diet (energy: 4.0 kcal g^−1^, 20% kcal from protein, 12% kcal from fat, 68% kcal from carbohydrates) and high-fat diet (energy: 4.7 kcal g^−1^, 16% kcal from protein, 42% kcal from fat, 43% kcal from carbohydrates).

**Table 2:** Primer sequences used for RT-PCR.

Genes	Primer Sequences	
	Forward (5′–3′)	Reverse (5′–3′)
β-actin	CTACCTCATGAAGATCCTGACC	CACAGCTTCTCTTTGATGTCAC
AMPK	CAACTATCGATCTTGCCAAAGG	AACAGGAGAAGAGTCAAGTGAG
PPAR-γ	TCCATTCACAAGAGCTGACCC	GGTGGAGATGCAGGTTCTACT
LXR-α	TGACTTTGCCAAACAGCTCC	TGTACCTCCGTGACGTCTC
SREBP-1C	CTGTGTGACCTGCTTCTTGT	CTCATGTAGGAACACCCTCC

**Table 3:** Body Weight for Experimental Mice.

Group	1	2	3	4	5	6	7	8	9	10
NC (g)	18.33 ± 1.05 ^a^	19.05 ± 0.94 ^a^	20.42 ± 0.70 ^b^	21.59 ± 0.84 ^b^	21.77 ± 1.75 ^c^	21.59 ± 1.2 ^b^	21.63 ± 0.57 ^c^	21.32 ± 0.71 ^c^	22.05 ± 0.77 ^b^	22.22 ± 0.64 ^c^
HFD (g)	18.29 ± 0.65 ^a^	18.77 ± 0.77 ^ab^	21.53 ± 0.52 ^a^	23.12 ± 0.60 ^a^	24.51 ± 0.39 ^a^	23.71 ± 0.81 ^a^	23.91 ± 1.05 ^ab^	24.04 ± 0.94 ^a^	25.15 ± 0.69 ^a^	26.59 ± 1.17 ^a^
JL1 (g)	18.25 ± 0.84 ^a^	18.44 ± 1.33 ^b^	21.34 ± 1.25 _a_	21.28 ± 1.30 ^b^	23.07 ± 1.12 ^b^	23.27 ± 1.42 ^a^	23.50 ± 1.56 ^ab^	23.38 ± 0.86 ^b^	22.83 ± 0.96 ^b^	23.78 ± 0.84 ^b^

Data are expressed as mean ± standard deviation (*n* = 8). Different letters denote significant differences between different groups of mice (*p* < 0.05).

**Table 4:** Effect of *L. rhamnosus* JL1 on the levels of serum lipids in mice.

Group	TC (mmol/L)	TG (mmol/L)	LDL-C (mmol/L)	HDL-C (mmol/L)
NC	3.85 ± 0.38 ^c^	1.34 ± 0.28 ^c^	0.51 ± 0.07 ^c^	4.74 ± 1.33 ^a^
HFD	6.09 ± 0.73 ^a^	2.02 ± 0.38 ^a^	0.98 ± 0.14 ^a^	3.20 ± 1.04 ^b^
JL1	4.98 ± 0.68 ^b^	1.69 ± 0.23 ^b^	0.69 ± 0.08 ^b^	4.30 ± 0.90 ^ab^

Data are expressed as mean ± standard deviation (*n* = 8). Different letters denote significant differences between different groups of mice (*p* < 0.05).

**Table 5:** Effect of *L. rhamnosus* JL1 on the levels of serum inflammatory cytokines in mice.

	IL-1β (ng/L)	IL-6 (ng/L)	IFN-γ (ng/L)	TNF-α (ng/L)
NC	16.98 ± 2.00 ^c^	45.76 ± 9.21 ^b^	185.86 ± 19.16 ^c^	187.20 ± 19.63 ^c^
HFD	30.23 ± 6.57 ^a^	69.55 ± 10.56 ^a^	284.30 ± 16.04 ^a^	297.54 ± 29.44 ^a^
JL1	24.17 ± 5.08 ^b^	49.48 ± 11.65 ^b^	227.80 ± 11.28 ^b^	257.60 ± 29.79 ^b^

Data are expressed as mean ± standard deviation (*n* = 8). Different letters denote significant differences between different groups of mice (*p* < 0.05).

## Data Availability

Data in the project is still being collected, but all data used in the study is available by contacting the authors.
